# Cortical traveling waves reflect state-dependent hierarchical sequencing of local regions in the human connectome network

**DOI:** 10.1038/s41598-021-04169-9

**Published:** 2022-01-10

**Authors:** Naoyuki Sato

**Affiliations:** Department of Complex and Intelligent Systems, School of Systems Information Science, Future University Hakodate, 116-2 Kameda-Nakano, Hakodate, Hokkaido 041-8655 Japan

**Keywords:** Computational neuroscience, Dynamical systems, Network models

## Abstract

Recent human studies using electrocorticography have demonstrated that alpha and theta band oscillations form traveling waves on the cortical surface. According to neural synchronization theories, the cortical traveling waves may group local cortical regions and sequence them by phase synchronization; however these contributions have not yet been assessed. This study aimed to evaluate the functional contributions of traveling waves using connectome-based network modeling. In the simulation, we observed stable traveling waves on the entire cortical surface wherein the topographical pattern of these phases was substantially correlated with the empirically obtained resting-state networks, and local radial waves also appeared within the size of the empirical networks (< 50 mm). Importantly, individual regions in the entire network were instantaneously sequenced by their internal frequencies, and regions with higher intrinsic frequency were seen in the earlier phases of the traveling waves. Based on the communication-through-coherence theory, this phase configuration produced a hierarchical organization of each region by unidirectional communication between the arbitrarily paired regions. In conclusion, cortical traveling waves reflect the intrinsic frequency-dependent hierarchical sequencing of local regions, global traveling waves sequence the set of large-scale cortical networks, and local traveling waves sequence local regions within individual cortical networks.

## Introduction

In the nervous system, neural oscillations are observed at various spatial and temporal scales^1,2^, including local field potentials, electrocorticography (ECoG), electroencephalography (EEG), and magnetoencephalography (MEG). The synchronization of oscillations in distant brain regions is considered essential for the implementation of brain functions^3–5^, such as perceptual binding^3^, discrete perception^6^, selective information transfer^4^, sequential memory^7^, and synaptic plasticity. In cross-frequency coupling^8^, phases of slower oscillation are locked to the amplitude of faster oscillations, providing an important clue for information processing in the brain. According to the communication-through-coherence theory^4^, the synchronization of slower (e.g., beta band, 15–25 Hz) oscillations of two regions with conduction delay achieves unidirectional communication between regions in the faster (e.g., gamma band, 30–100 Hz) oscillations. Therefore, neural oscillations and their phase patterns in large-scale networks are critical to understanding the integrative processing of distributing functions in the brain.

EEG has demonstrated that alpha and theta band oscillations form traveling waves on the scalp during a variety of cognitive states, such as sleep^10^, resting^9^, auditory^11^ and visual processing^12–15^, memory retrieval^16^, and free viewing^17^. In addition to traveling waves in local brain regions on the millimeter scale^18–20^, recent human ECoG studies^22–26^ have also demonstrated that traveling waves with oscillations in lower frequency bands (alpha and beta bands) were also evident on cortical surfaces (gyri). The ECoG traveling waves, measured from a broad cortical area with high spatial resolution, are valuable to the understanding of integrative processing in the brain. According to neural synchronization theories, traveling waves group and sequence local regions in the entire network. Such global functioning is associated with cortical resource allocation, particularly for complex tasks like literature understanding^27,28^. However, these associations have not yet been experimentally evaluated possibly because ECoG measurements usually involve the gyri rather the sulci, and the whole brain network cannot be evaluated by this method alone.

In early theoretical works^29,30^, the modeling of cortical tissue mass, including the number of active excitatory and inhibitory synapses, has been successfully predicted using cortical traveling waves; however, these predictions did not explain the detailed function of traveling waves as they relate to local, individual regions. More recently, researchers have developed the connectome-based modeling approach^31–34^ based on data-driven structural connectivity and biologically plausible neural population dynamics. This approach is considered useful for the evaluation of cortical traveling waves and their potential functions. Connectome-based models have successfully simulated experimental observations, including resting-state functional connectivity^35,36^, metastability with higher (gamma) oscillations^37,38^, resting state MEG connectivity^39^, metastability of MEG^40^, and scalp EEG topography^41^. Some of the models also simulated traveling waves on the cortical surface^34,40^; however, their functional contributions have not been evaluated. These reports confirm the availability of connectome-based modeling for the evaluation of traveling waves with biological plausibility.

**Figure 1:**
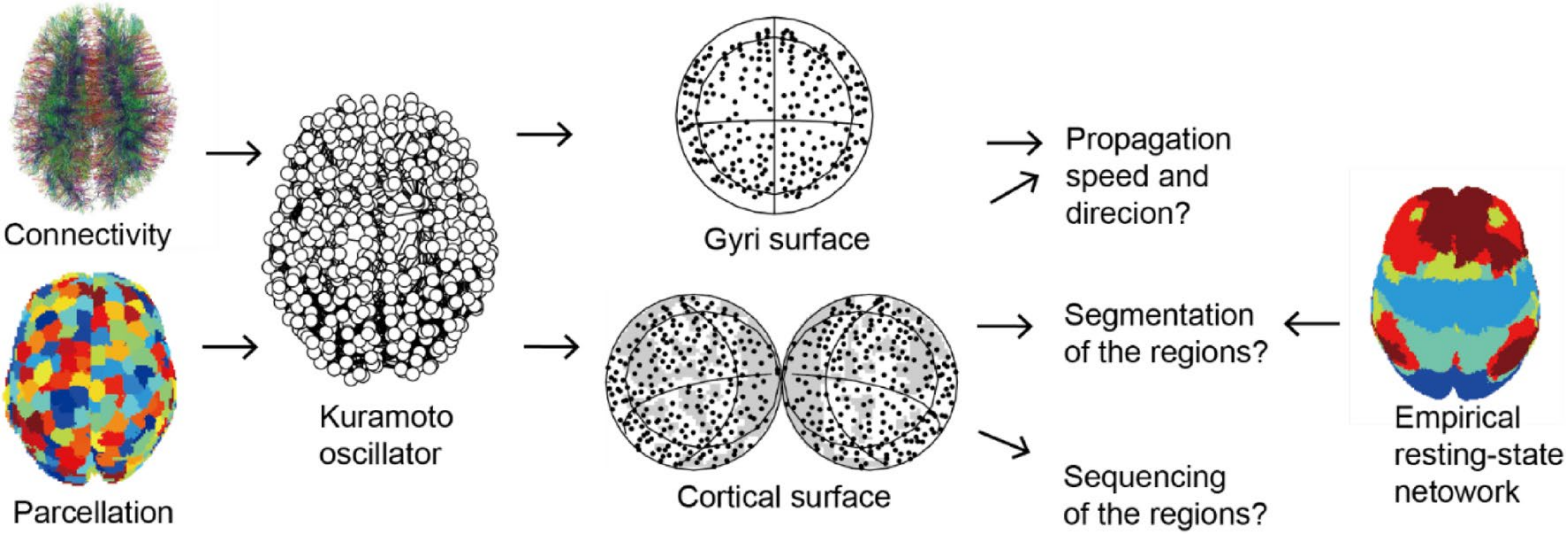
Analysis procedure. A network model using the Kuramoto oscillator is constructed based on parcellation and connectivity datain humans. Traveling waves in the model are evaluated by comparing them to the empirically observed resting-state networks. Connectivity image on the upper left was generated using DTI studio software (Mar 5, 2021 build; http://dsi-studio.labsolver.org).

This study aimed to evaluate the functional contribution of cortical traveling waves using connectome-based network modeling (Fig. 1). We evaluated the potential functions of traveling waves using the following two approaches: (1) the grouping of local cortical regions into large-scale networks and (2) the sequencing of these local regions. In terms of model construction, traveling waves can be generated by multiple neural mechanisms^42^. However, the model applied in this study was proposed as a large-scale coordination mechanism for ECoG traveling waves^24^, and was based on the theory of coupled phase oscillators^42^ wherein weakly coupled oscillators with different intrinsic frequencies produced traveling waves, i.e., synchronized oscillation with a time lag. To implement the mechanism, we used the Kuramoto model^43,44^ to represent neural dynamics in local cortical regions which focuses on describing phase dynamics, and is comparable with numerous other connectome-based models^35–37,39,45^.

## Results

**Figure 2:**
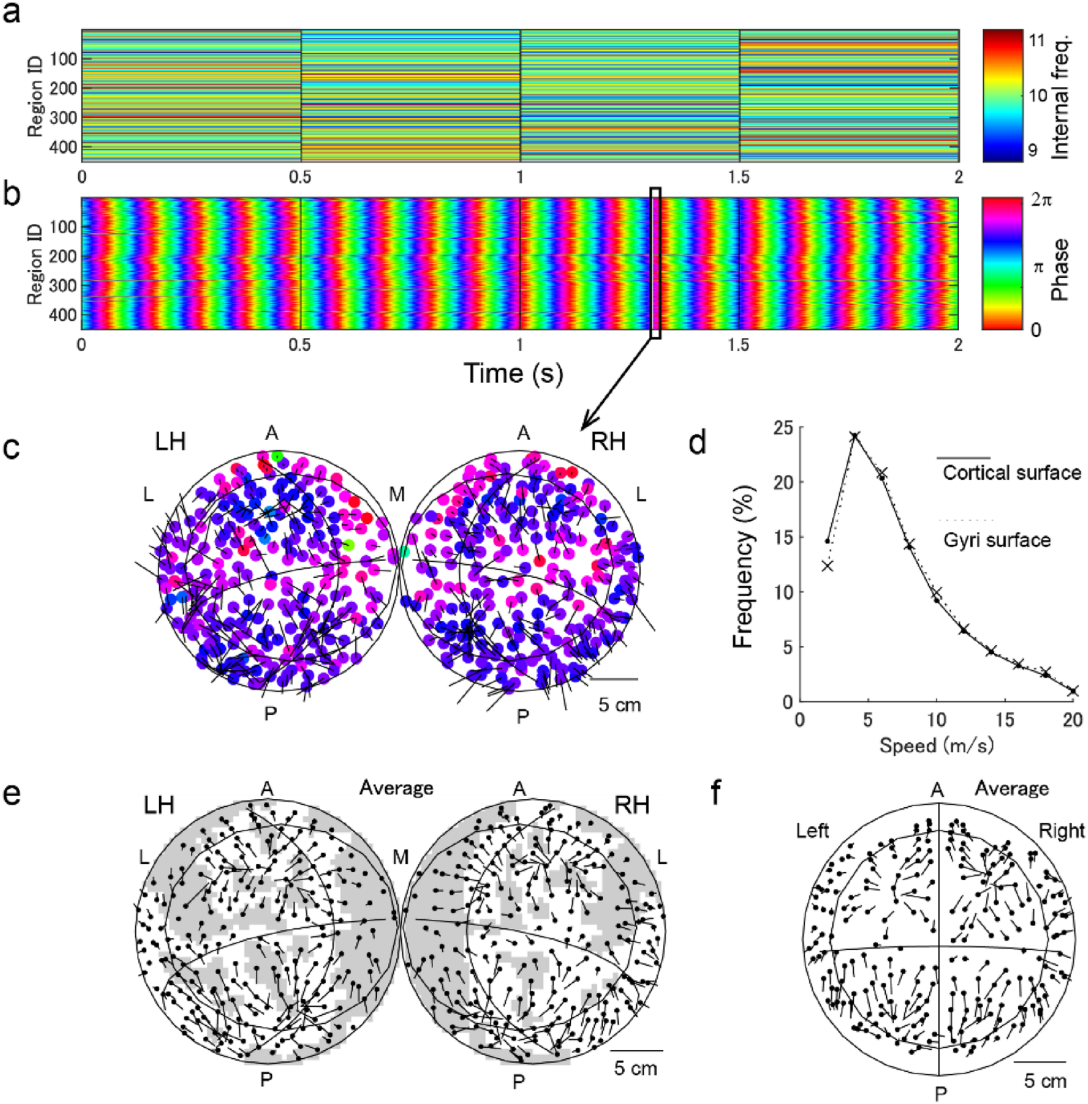
Traveling waves. (**a**,**b**) Time series of internal frequency and phase in each region. (**c**) Cortical surface map of instantaneous phases ($t$=1.3 s). The filled circles denote the locations of regions and the colors represent their phases. The large circles represent each hemisphere and the lines display the abstract correspondence to the axes in the Montreal Neurological Institute (MNI) space; ellipse denotes $z$ =0, the horizontal curveed lines denote $y$ = − 24, and the vertical curved lines denote $x$ = $\pm$ 10. A and P denote the anterior and posterior sides, respectively. L and R denote the left and right sides, respectively. M denotes the medial side. LH and RH denote the left and right hemispheres, respectively. Arrows pointing outward from each node depict the phase gradient from the early to late phases, and the length of the arrow is proportioanal to its velocity. (**d**) The distribution of propagation speed over 200 s. (**e**) Cortical surface map displaying the average direction of traveling waves. The gray shading shows the areas in the sulci. (**f**) Gyri-surface map displaying the average direction of traveling waves, where the inner circle, horizontal and vertical lines represent axes of $z$ =0, $y$ = − 24 and $x$ =0, in the MNI space, respectively.

In the simulation, 468 brain regions of the thalamocortical network with appropriate conduction delays were connected in relation with human connectome data (Fig. 1). Traveling waves on the cortical surface were evaluated on a map that included both hemispheres with unfolded sulci. We performed the simulation for 100 s while varying internal frequencies of the regions (mean ${f}_{0}$=10 Hz; standard deviation ${\sigma }_{f}$=0.5 Hz) at 0.5 s intervals (Fig. 2a,b); the simulation data were used in the analyses that followed.

### Generation of traveling waves

In the simulation, the wave propagation was stable (Fig. 2c; also see Supplementary Video 1) and the propagation speed was within a biologically plausible range of an axonal conduction speed of 1–10 m/s^46^ (median, 6.5 m/s; IQR, 4.1–11.0 m/s; Fig. 2d). The simulated propagation speeds closely corresponded with propagation speeds on the gyri surfaces (median, 6.7 m/s; IQR, 4.3–11.2 m/s). The similarity was thought to be related more with the long wavelengths than any individual sulci, as also demonstrated by a previous EEG study^15^. The average direction of wave propagation (Fig. 2e,f) displayed sinks of propagation in the bilateral frontal and occipital areas, which is consistent with a previous report on connectome-based modeling^40^.

### Grouping of regions by traveling waves

**Figure 3:**
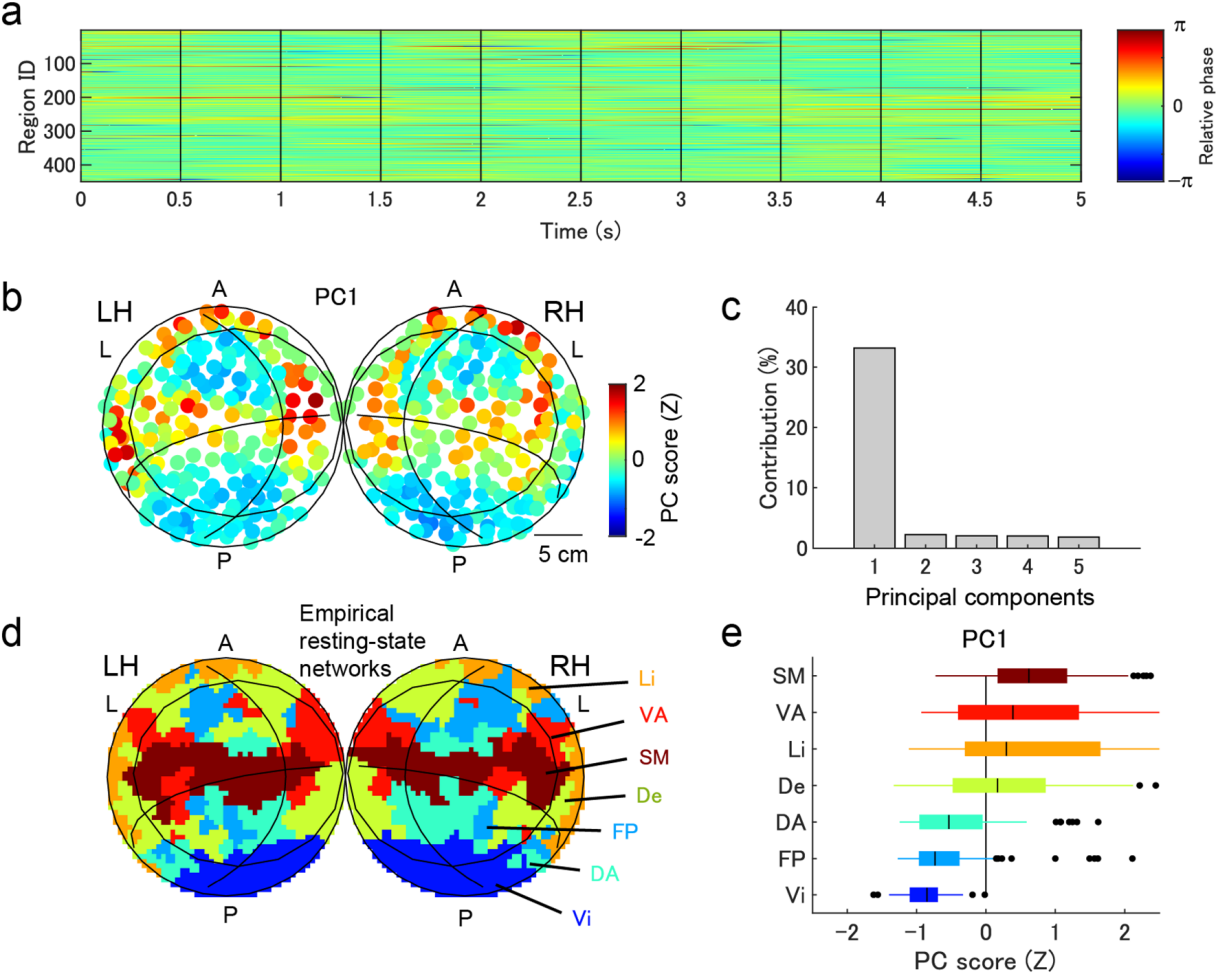
Relative phases. (**a**) Time series of the relative phase. (**b**) Cortical surface map representing the first principle component of the relative phase. (**c**) Contributions of principal components of the relative phase. (**d**) Cortical surface map displaying the empirically observed resting-state networks^47^. *SM* somatomotor network, *VA* ventral attention network, *Li* limbic network, *De* default network, *DA* dorsal attention network, *FP* frontoparietal network, *Vi* visual network. (**e**) Phase distribution in each large-scale network. Points represent outliers defined by data outside of [Q1 − 1.5 *IQR, Q3 + 1.5*IQR].

We assessed the topographic pattern of phases, considered important for the segregation of networks based on temporal binding, by comparing the relative phases that were temporally stable with the phase values (Fig. 3a). Figure 3b depicts the first principal component of the relative phase, where the first principal component was dominant (33%) compared with other components (Fig. 3c). Interestingly, this pattern correlated with that of the resting-state network from functional magnetic resonance imaging (fMRI)^47^ (Fig. 3d). Phase distributions of regions included in the empirical networks (Fig. 3e) also revealed a significant inter-network difference in the phases (F(6,443) = 37.3, p < 1e−6).

**Figure 4:**
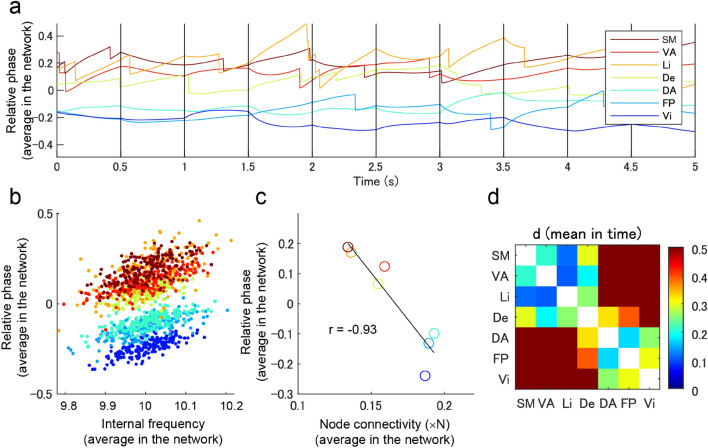
Instantenous grouping of regions in the large-scale networks. (**a**) Time series of relative phases averaged in each network. (**b**) Relationship between the internal frequency (averaged in the network) and the relative phase (averaged in the network) and (**c**) relationship between the relative phase (averaged in the network) and the node degree (averaged in the network). The colorcode is identical with those in (**a**,**c**). (**d**) Effect size of phase difference between the paired networks.

We further evaluated the network segmentation effect by analyzing the relative phases in the time series. Average phases of regions belonging to the empirical networks (Fig. 4a) were quickly re-ordered according to changes in the internal frequencies of the local regions (with a 0.5 s interval). The average phases appeared to be a function of the average internal frequencies of regions included in each empirical network (Fig. 4b), where the bias of the average phase was significantly correlated with the average node degree of the regions belonging to the empirical network (Fig. 4c; r = − 0.93; t(5) = 10.97; p = 1.1e−4). We measured the phase differences of individual network pairs in the empirical networks by the effect size (Fig. 4d), where the difference of 17 pairs was considered more than small (0.2) except for the following four pairs: somatomotor and limbic networks, ventral attention and limbic networks, ventral attention and default networks, and dorsal attention and frontoparietal networks. An additional permutation test on the phase difference between the paired networks (with multiple comparison correction by false discovery rate < 0.05) also demonstrated that the difference between the 17 pairs was significantly larger than zero (p < 0.05).

### Sequencing of regions by traveling waves

**Figure 5:**
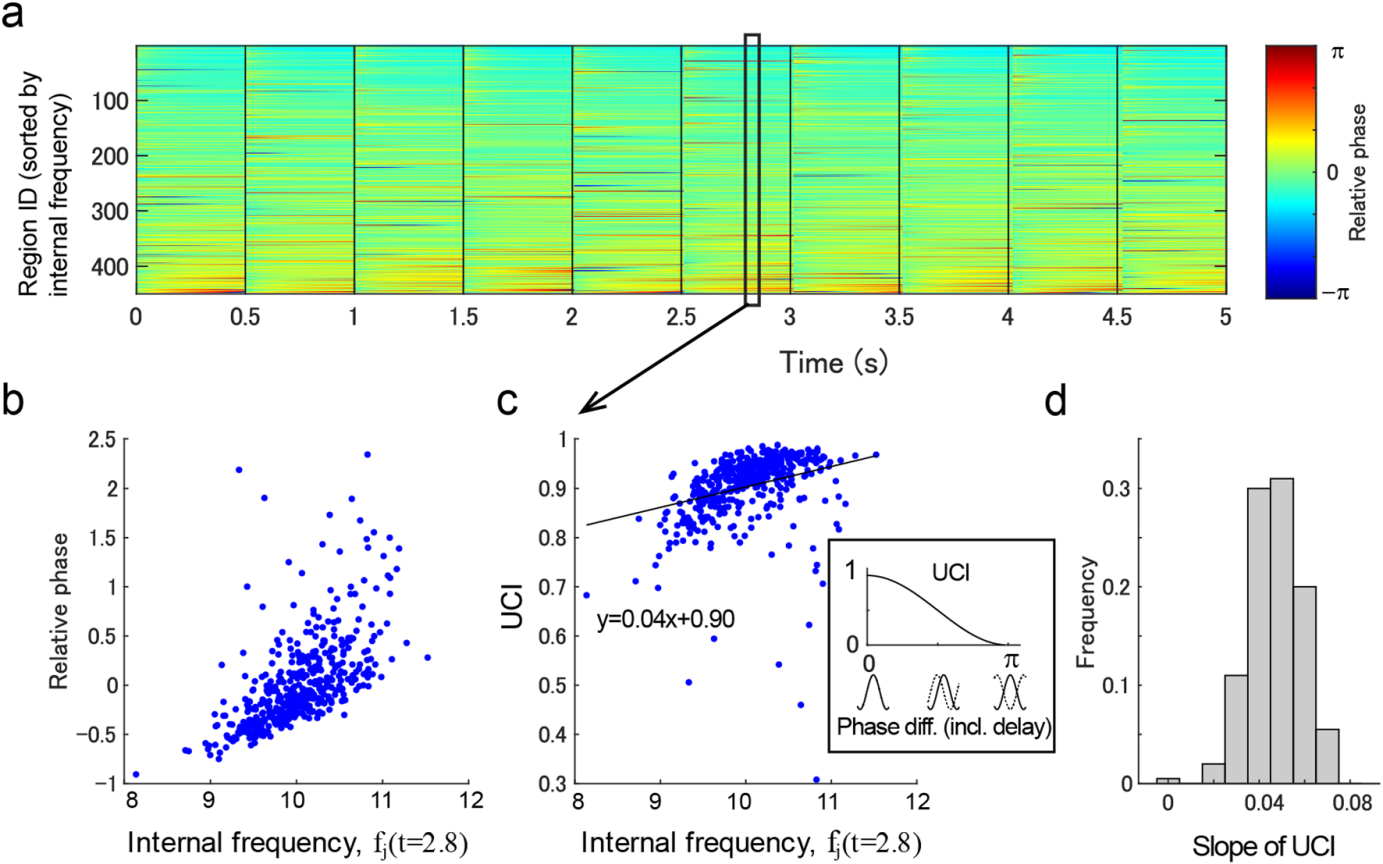
Dependence of phases on internal frequencies. (**a**) Time series of relative phases (data identical to Fig. 3a but sorted by the internal frequency of the region). (**b**) Relationship between the internal frequency and relative phase at $t$=1.3 s. (**c**) Unidirectional communication index (UCI) calculated from the data presented in (**b**). (**d**) The distribution of the slope of UCIs measuring the internal frequency dependence of unidirectional communication.

We further evaluated the phase structures among the individual regions in relation with their internal frequencies. Interestingly, the relative phases were instantaneously ordered in the entire network according to their internal frequencies (Fig. 5a,b). Based on the communication-through-coherence theory^4^, phase difference and conduction delay between the regions can cause unidirectional communication. Therefore, we defined the unidirectional connectivity index (UCI), which represented the degree of outward communication from the region (Fig. 5c). Figure 5c depicts that the relative phases as correlated with the internal frequency (Fig. 5b) were within the temporal range producing unidirectional communication. We further defined the so-called sequencing effect as the slope of UCI to the intrinsic frequency used to measure the organization of the entire network with directional communication. By this definition, a larger positive slope indicated more effective information transfer from regions with faster intrinsic frequencies. In the analysis of all-time series, the slope of UCI was significantly positive (Fig. 5d; mean of the slope of UCI = 0.05; t(199) = 59.7; p < 1e−6).

**Figure 6:**
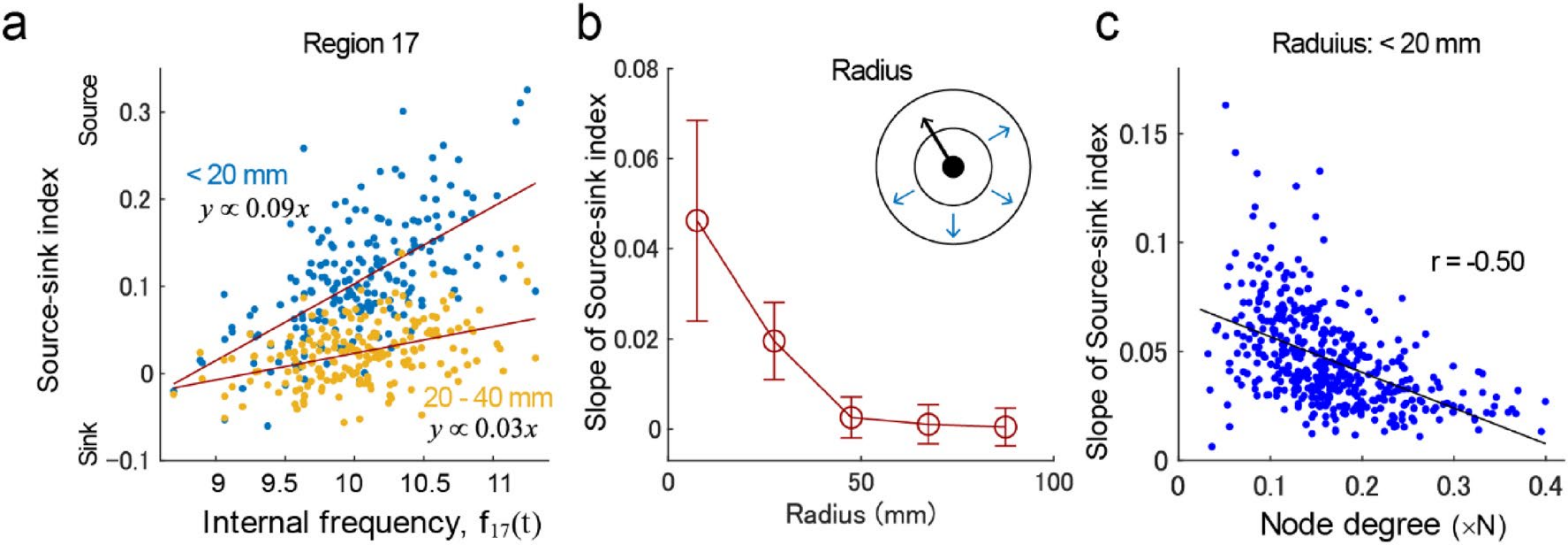
Local radial waves. (**a**) The relationship between the internal frequcncy and the source-sink index (SSI) (region 17). (**b**) The spatial extent of internal frequency-dependent radial waves, measured by the slope of SSI. (**c**) The relationship between the node degree and the slope of SSI.

Investigating the process of integrating the sequencing effect in individual regions with the network segmentation effect based on topographically traveling waves was warranted. To address this question, we evaluated radial waves generated locally from a region (the phase structure termed “phase cone”^48^, originally reported on a scale of several millimeters) with an index defined by the degree of outward (or inward) waves at a radius from the region (i.e., source-sink index [SSI]). Figure 6a depicts the SSI for region 17 (node degree, 0.09) which indicates that a faster intrinsic frequency is associated with stronger outward waves. Here, the slope of SSI indicated the dependence of the internal frequency on local waves. In the population analysis, the slope of SSI was large (< 50 mm; Fig. 6b), and its size was consistent with that of the wave cluster in the previous ECoG study^24^. The slope of SSI was larger for regions with a smaller node degree (i.e., non-hub regions; Fig. 6c), thereby indicating that the local waves depended on the connection structure.

### Relationship between state-dependent global and local waves

**Figure 7:**
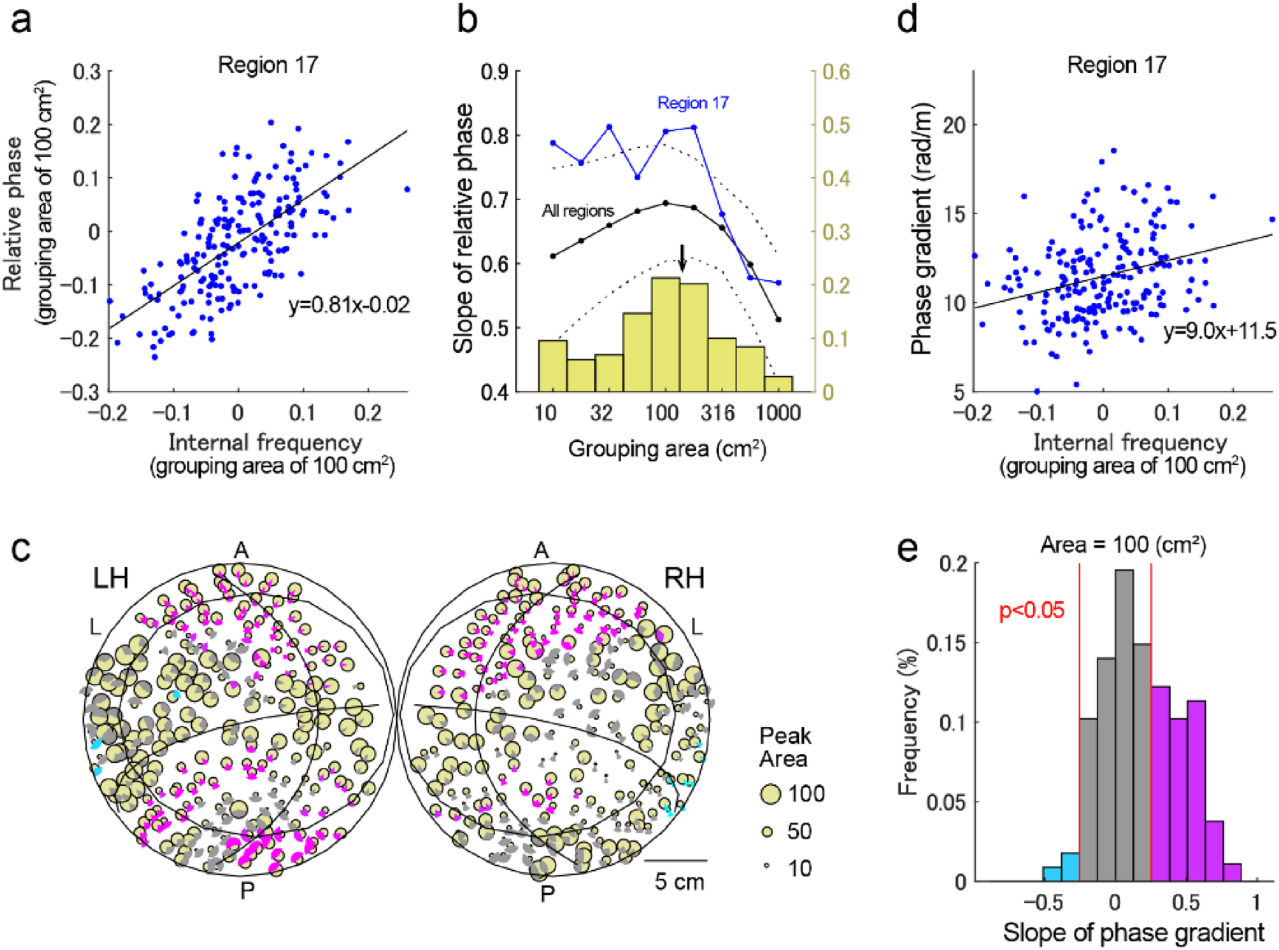
Influence of grouping size on internal frequency-dependent phase gradients. (**a**) Relationship between the internal frequency and the relative phase (grouping area of 100 cm^2^). (**b**) Relationship between the grouping size and the slope of the regression between the internal freqency and the relative phase. The blue line denotes the slope of the region at the center of region 17. Black solid and dashed lines denote the mean and the standard deviation of the slope of regions at every location. Yellow bars denote the histogram of the optimal size producing the maximum slope. The arrow indicates the mean size of the empirical resting-state networks. (**c**) A cortical surface map of optimal area sizes leading to the maximum slope of the relative phase (yellow circles). Gray sector disks represent the distribution of the direction of traveling waves (mean ± standard deviation) and their colors (magenta and cyan) denote the slope of internal frequency to phase gradients (positive and negative correlation, respectively; corresponding to **d**; grouping area of 100 cm^2^). (**d**) Relationship between the internal frequency and phase gradient of the traveling wave (grouping area of 100 cm^2^). (**e**) Distribution of the slope of the regression between the internal frequencies and the phase gradients. Magenta and cyan bars show significant values of slopes and red lines denote the statistical thresholds of p < 0.05 (permutation tests, 2000 times).

What is the relationship between the following two types of traveling waves: the type associated with the phase gradient in the entire network (Fig. 5b) and the type showing a local phase cone structure (Fig. 6)? To understand this relationship, the influence of the grouping size on the regression between internal frequency and the local regions to their relative phases was evaluated using circular areas for the grouping of local regions by varying their radii. Figure 7a shows the regression from internal frequency to relative phase with a grouping area of 100 cm^2^ at the central location of region 17. When the grouping size was varied, a grouping size of 100 cm^2^ was found to maximize the influence of the internal frequency on the relative phases (Fig. 7b). Figure 7c shows a cortical surface map of the optimal grouping sizes for maximal influence, wherein the spatial pattern was significantly correlated with that of the empirically resting-state networks (Fig. 3c; F(6, 499) = 8.45; p < 1e−8). Moreover, spatial phase gradients at each location were also dependent on the internal frequencies of the grouped regions (Fig. 7c), wherein the spatial pattern was also significantly correlated with that of the empirical networks (Fig. 7d,e; F(6, 499) = 10.32; p < 1e−10). Importantly, the optimal grouping size of 100 cm^2^ (radius 56 mm) producing the maximal influence on the relative phase agreed with both the size of the resting-state network (140 ± 52 cm^2^) and the size of the local phase cone (< 50 mm; Fig. 6). This finding suggests that the global traveling waves (over the entire cortex) function in the sequencing of large-scale functional networks, and the local traveling waves (forming phase cones) function in the sequencing within the size of individual networks.

### Dependence of model parameters

**Figure 8:**
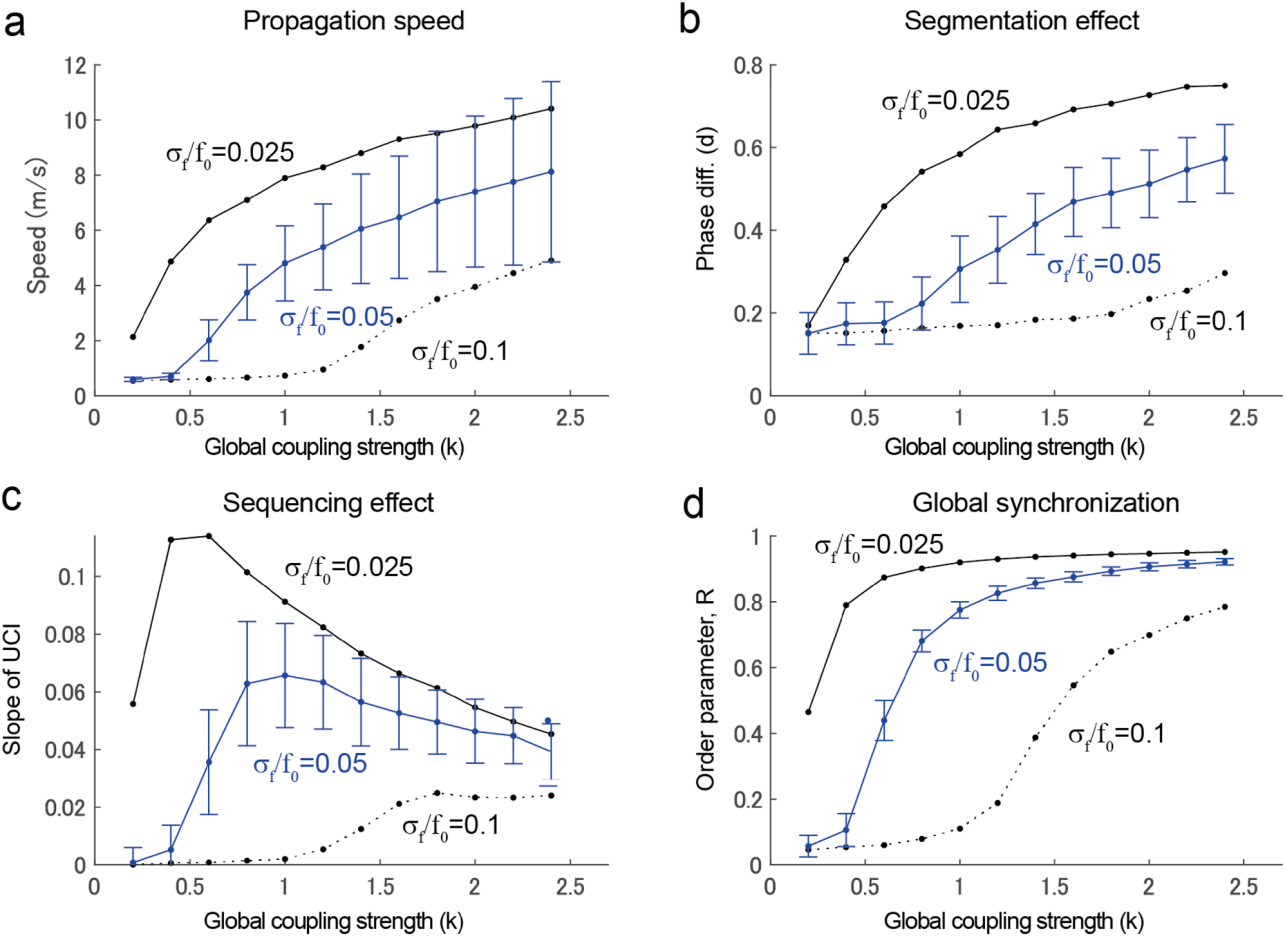
Parameter dependence of model behaviour. (**a**) Velocities of traveling waves. (**b**) Segmentation effects measured by the effect size of the phase difference between networks (Cohen’s d). (**c**) Sequencing effects measured by the slope of UCI. (**d**) Order parameter displaying synchronization of the entire network. Black, blue, and black dotted lines denote the parameters $\frac{{\theta }_{f}}{{f}_{0}}$= 0.025, 0.05, and 0.1, respectively. Errorbars denote standard deviation, but these were only displayed in cases of $\frac{{\theta }_{f}}{{f}_{0}}$ =0.05.

**Figure 9:**
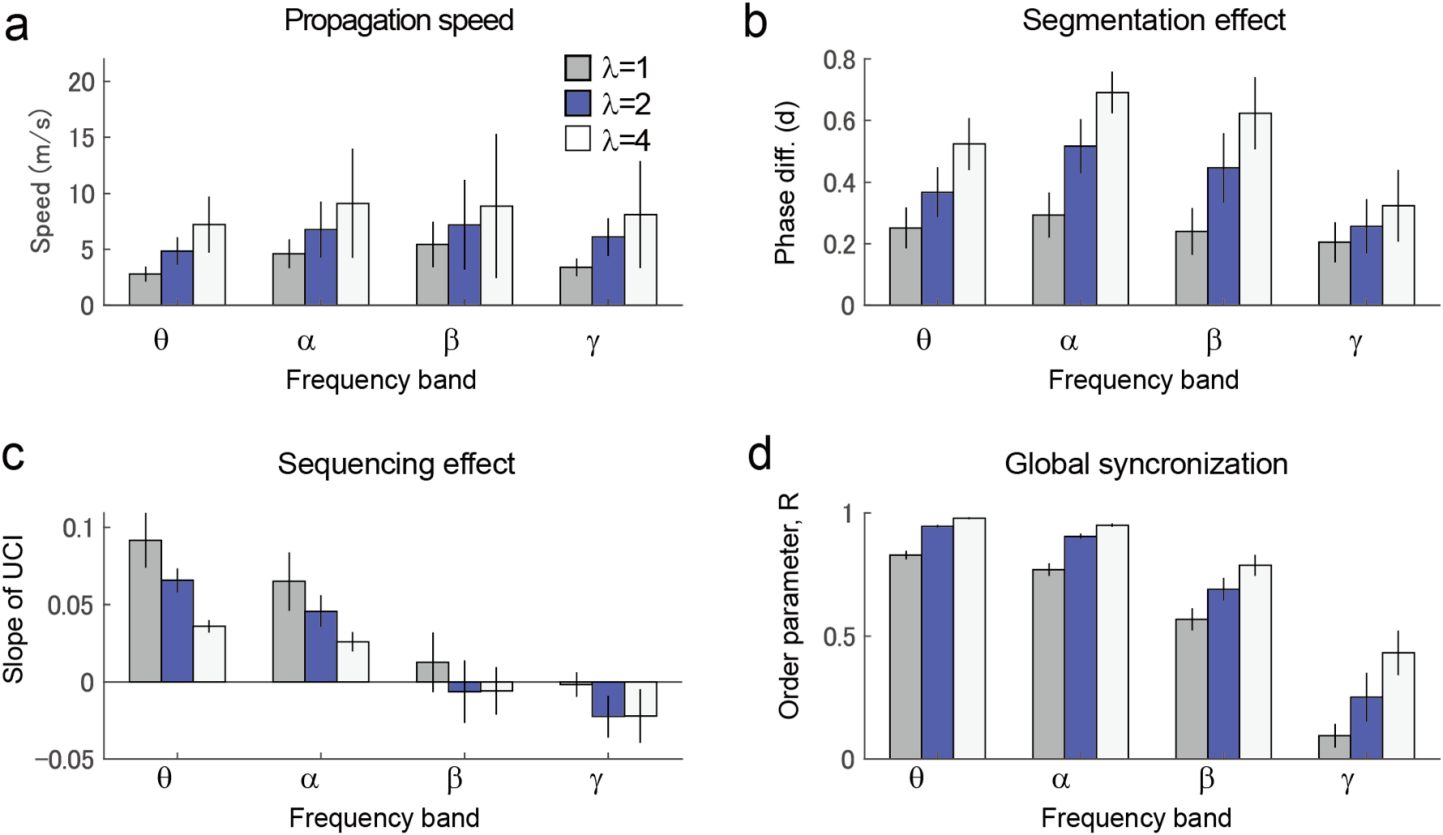
Dependence of parameters on frequency. Simulation of each osillation frequency was performed using the mean internal frequency ($${f}_{0})$$ of 5 (theta band), 10 (alpha band), 20 (beta band) and 40 (gamma band) Hz, with a constant standard deviation of $$\frac{{\theta }_{f}}{{f}_{0}}$$=0.05. (**a**) Velocities of the traveling waves. (**b**) Segmentation effect measured by the effect size of the phase difference in networks. (**c**) Sequencing effect measured by the slope of UCI. (**d**) Order parameter, R.

Finally, we tested the parameter dependency of our findings by varying the global coupling strength ($k$) and the standard deviation of the internal frequency (${\sigma }_{f}$; Fig. 8). The propagation speed (Fig. 8a), network segmentation effect (Fig. 8b), sequencing effect (Fig. 8c), and global synchronization (measured by the order parameter $R$) were found to be relatively stable in the neighboring parameters used in the simulation ($k$=2; ${\sigma }_{f}$=0.05). Interestingly, there was a trade-off between the segmentation and sequencing effects. In addition, the necessity of slow oscillation in the segmentation and sequencing effects was evaluated by varying the mean internal frequency (${f}_{0}$; Fig. 9). While the segmentation effect was notably stable within a broad range of frequency bands (theta, alpha, and beta), it was restricted in the lower frequency group (theta and alpha).

## Discussion

We first evaluated the possible functional contribution of traveling waves on the entire cortical surface using connectome-based modeling. In line with neural synchronization theories^3–5^, the travelling waves were confirmed to be functional; however, this study is the first to demonstrate the hierarchical formation of state-dependent sequencing of local regions, that global traveling waves reflect the sequencing of individual empirical resting-state networks (Fig. 4), and that local traveling waves (forming phase cone^48^ structures) reflect the sequencing of local regions within the grouping size of individual networks (< 50 mm) (Figs. 6 and 7). In the finding of the current study, the emergence of correlated activities in the empirical networks (Fig. 3) agree with the findings of previous modeling studies^35–37,49^. However, the current study further demonstrated that local regions in the entire network can be instantaneously sequenced depending on their states, and the sequence was expected to facilitate asymmetric communication between the regions^4^ (Fig. 5).

In the current simulation, traveling waves corresponded well with previous experimental and computational reports. The propagation speed (median, 6.5 m/s; IQR, 4.1–11.0 m/s; Fig. 2d) was within biologically plausible range of an axonal conduction speed of 1–10 m/s^22^; however, the speed was moderately faster than ECoG observations at 2–5 m/s^18,26^ or < 2 m/s^23–25^. The spatial pattern of traveling waves (Fig. 2e) was consistent with the pattern in a previous modeling report^40^, in that both reports demonstrated that the frontal and lateral parietal regions appeared as sinks of traveling waves. Our findings may also agree with ECoG observations displaying waves from the anterior to posterior regions^23,24^; however, the direction of traveling waves can vary^25^. Moreover, the sizes of local waves in the current study were < 50 mm, consistent with the spatial distribution of ECoG signals^24,50^. These correspondences would support the current simulation as plausible for the generation of traveling waves.

A major aspect of the current report was that the dominant pattern of traveling waves corresponded well with empirically observed resting-state networks (Fig. 3). However, there was flexibility in the order of phases of individual networks depending on the internal frequency of the networks (Fig. 4). Another aspect of the current report was that instantaneous traveling waves contributed to sequencing of entire local regions in the order of their internal frequencies (Fig. 5), which is consistent with the theory of a coupled phase oscillator^42^ wherein weakly coupled oscillators with different intrinsic frequencies synchronize with a lag in relation to the frequency difference. The importance of this result is that the sequencing effect occurs in the entire network with biologically plausible connectivity. Moreover, the phase distribution appeared to support unidirectional communication between these regions based on the communication-through-coherence theory^4^. This instantaneously formed phase structure is a derivative of the hierarchical structure that organized the processing of the entire cortex, which may be required for resource allocation. The sequencing effect was combined with local radial waves (Fig. 6). The size of local waves in the current study was < 50 mm, which is consistent with the spatial size of individual large-scale networks and with the grouping size that produced the maximal influence of the internal frequency on the relative phases (Fig. 7). This result suggested a hierarchical sequencing of local regions in the entire network, that the information of local regions was integrated by the local traveling waves, and the information of the individual large-scale networks was integrated by the global waves.

Hub regions with dense connectivity to others region play dominant roles in the organization of effective connectivity^33,36,40^. In the current simulation, these regions were considered to play an important role in synchronizing the global network. However, the contribution of hub regions in the formation of traveling waves was not strongly emphasized. This is because large-scale networks, including regions with a larger node degree, appeared in the later phase (Fig. 4c); therefore, the findings indicate that these networks tend to receive information from other networks based on the communication-through-coherence theory^4^. Moreover, the local generation of radial waves is weak in hub regions when compared with non-hub regions (Fig. 6c). In other words, the hub regions may play a role in synchronizing large-scale networks and integrating information by receiving it from surrounding regions.

The current results support the proposal of two views for process integration by traveling waves. One, the cortical traveling waves can be considered as a kind of central pattern generator (CPG)^51,52^, a concept that was originally proposed as a mechanism of generating locomotion or respiration patterns of neural networks. The functional patterns are generated by the intrinsic property of the network and dynamically modulated to associate its function with external inputs. The current study demonstrated that the cortical traveling wave can act as a CPG with the dominant spatiotemporal pattern that is flexibly modulated by local regions. While the cortical CPG is considered plastic^53^, the current proposal is based on the contribution of traveling waves to information processing on the entire cortical surface. Second, the traveling wave acts as a kind of medium for integrating local information. This information produces local waves of < 50 mm, and the state of the entire network is represented as a wave pattern, such as when water droplets disrupt the still surface of water in a jar. The hierarchical sequencing of the entire network by traveling waves likely contributes to the solution of global optimization problems. Both views potentially support the understanding of large-scale network coordination, and will likely give some insight into the biologically plausible implementation of the unified brain theory^54^.

This study had several limitations. First, traveling waves in the current simulation were stable for the provided internal frequencies; however, they spontaneously fluctuated during experimental observation. In previous modeling studies^37–39^, gamma-band oscillations played a critical role in producing fluctuation in slower oscillations. This necessitates the inclusion of cross-frequency coupling for a complete understanding of traveling wave dynamics, as is applied in connectome-based modeling^55^, for a complete understanding of the dynamics of traveling waves. Second, the thalamus in the current model was homogeneous with the cortical network; this is also the case in previous connectome-based models^35,36^. The thalamus plays a critical role in cortical wave propagation^18,23^; however, in the current simulation, exclusion of the thalamus (18 regions) did not result in a significant change in the results (data not shown). Unidirectional interactions in the thalamocortical network^56^ or differentiated dynamics to the cortex are considered essential for future investigations. Third, traveling waves in scalp EEG^9,10^ were not included in the current analysis. While some connectome-based modeling evaluated scalp EEGs^41^, cortical folds and volume conduction produced complicated correspondence to scalp EEG findings, particularly for the traveling waves. From an application point of view, the detection of cortical propagation waves by analyzing scalp EEGs is significant; thus, it is regarded as an important consideration for future research. Finally, internal frequencies of local regions were reported using normally distributed random values; however, these values would be generated by spatial structures during cognitive tasks. Inclusion of such spatially structured internal frequencies may produce dynamic changes of traveling wave patterns, as observed in ECoG^24,25^ and EEG studies^13,16^. Inclusion of neural representation^57,58^ and its flow^21^ over the distributed cortical areas is also important for the complete understanding of the function of traveling waves during cognition.

## Methods

We evaluated traveling waves in terms of the grouping and sequencing of cortical regions using Kuramoto oscillators in a connectome-based network model. The model was nearly identical to previous models^35,37^ in terms of its usage, continuous connectivity, distance-related delay, and zero-noise to the phases. However, the current model differed from previous models in the following two ways: the frequency band was moved from the gamma to the alpha band oscillation, and the internal frequencies of the oscillators were intermittently changed.

### Model

We determined network connectivity using the human connectome data. Regions of interest (ROIs) were generated by random parcellation of gray matter in the ICBM152 structural template^59^, in which 468 contiguous volumes of the cortex and thalamus were defined by k-means clustering under the constraint of the Automated Anatomical Labeling Atlas 3 (AAL3) template^60^ (mean volume, 2144 mm^3^; standard deviation, 399 mm^3^; range, 753–3077 mm^3^). In contrast with previous connectome-based models^36,37^, the thalamus was included in the network^34,39,40^, because the corticothalamic interaction was thought to be essential for the generation of traveling waves^18,23^. Connectivity of the ROIs was determined by open access diffusion MRI data (WU-Minn HCP1065, 1 mm^61^)), wherein the individual connectivity of 1,065 subjects was calculated using DTI studio (Mar 5, 2021 build; http://dsi-studio.labsolver.org; q-space diffeomorphic reconstruction^62^, 5 × 10^6^ seeds) and the numbers of streamlines were averaged to obtain a structural connectivity matrix and a tract length matrix ($L$). The weights, divided by the stream length^63^, were normalized by the volume of each region^64^. We calculated the propagation delay matrix ($D$) under a fixed velocity assumption as $D=L/v$ with a velocity $v$, which led to a mean $D$ of 6 ms^35^ (($v$ =10.8 m/s, mean $L$=108.3 mm). All methods were performed in accordance with the WU-Minn HCP guidelines (https://www.humanconnectome.org/study/hcp-young-adult/document/wu-minn-hcp-consortium-open-access-data-use-terms).

The oscillation in each region was described by a phase oscillator displaying self-sustained oscillation. The network of connected oscillators was calculated according to the following equation:

$$\frac{d{\theta }_{j}(t)}{dt}={\omega }_{j}\left(t\right)+\lambda \sum_{k=1}^{N}{w}_{jk}\mathrm{sin}({\theta }_{k}\left(t-{\tau }_{jk}\right)-{\theta }_{j}\left(t\right))$$

where ${\theta }_{j}$ and ${\omega }_{j}$ are the phase and internal angular frequency of the oscillator $n$, respectively, λ is the global coupling strength, and ${w}_{jk}$ and ${\tau }_{jk}$ are the weight and conduction delay between oscillators $j$ and $k$. The internal frequency ${f}_{n}={\omega }_{n}/2\pi$ was produced by a fixed Gaussian distribution with mean ${f}_{0}$ and standard deviation ${\sigma }_{f}$(${f}_{0}$=10 Hz and ${\sigma }_{f}/{f}_{0}$=0.05 here). Simulations were run for 101 s by changing the internal frequencies with 0.5 s intervals. Relative phases were generated by the following equation:

$${\theta }_{j}^{^{\prime}}={\theta }_{j}-arg\sum_{k=1}^{N}{e}^{i{\theta }_{k}};(-\pi , \pi ]$$

where $arg x$ denoted the argument of the complex value $x$, and appeared continuous in time. We analyzed 200 time points sampled at 0.3 s following each change. The first 1 s was discarded from the analysis.

### Evaluation of wave propagation

Spatial patterns of instantaneous phases were evaluated using two-dimensional maps as follows: the cortical surface map and the gyri-surface map. Each map was generated by the Lambert's azimuthal equal-area projection of the ROI locations on a spherical surface. The cortical surface map was calculated from two inflated spherical surfaces of both hemispheres, calculated using FreeSurfer (https://surfer.nmr.mgh.harvard.edu/) with the polars provided by the C1 and C3 locations in the 10–20 system^65^. The gyri surface map was calculated from a spherical surface comprising projections from 230 regions of the gyrus (61.3% of all regions), with the polarity of the Cz location. Superficiality was defined by the distance of regions from a hypothetical mid-surface (the averaged surface points in a range of 15 mm radius), with a standard deviation > 0.5. In these maps, the shapes of local regions were distorted, particularly in the bottom face of the brain; however, the advantages of these shapes was in their continuity over the entire surface with homogeneously dense regions.

We evaluated the spatiotemporal pattern of phases in these surfaces using the following features:

#### The velocity of wave propagation

Instantaneous phase gradients were calculated using a circular-linear regression^66^ with a Gaussian window of size 20 mm, followed by calculating the velocities of contours of the constant phase^20^.

#### Topographical components of phases

Relative phases were more stable than instantaneous phases and were used to characterize the phase pattern^11,12,15^. We extracted spatial components of the patterns in the relative phases by principal component analysis (PCA) because the distribution of the phase (< π) demonstrated a single-peaked distribution similar with a Gaussian distribution.

#### Phase-based segmentation associated with empirical resting-state networks

Similar phases forming a band in the traveling waves supposedly contributed to the segmentation of local networks. This ability was evaluated using the phase difference of regions in the empirically observed resting-state networks^47^, which included seven network types (visual, somatomotor, dorsal attention, ventral attention, limbic, frontoparietal, and default networks). We measured the differences in phases between the two networks $i$ and $j$ using the effective size (or Cohen’s d) as follows:

$${d}_{ij}\left(t\right)=\frac{|mean\left({P}_{i}\left(t\right)\right)-mean\left({P}_{j}\left(t\right)\right)|}{\sqrt{\frac{(\left({N}_{i}-1\right)var\left({P}_{i}\left(t\right)\right)+\left({N}_{j}-1\right)var({P}_{j}\left(t\right))}{({N}_{i}+{N}_{j})}}}$$

where ${P}_{i}\left(t\right)$ denotes a set of regions’ phases (at time $t$) in a network $i$, ${N}_{i}$ denotes the number of regions in the network $i$, and $mean(x)$ and $var(x)$ denote the mean and variance of $x$, respectively. The relevant sizes of d were proposed as small (0.2), medium (0.5), and large (0.8).

We evaluated the influence of internal frequencies to individual regions using the following three measures:

#### Unidirectional communication index (UCI)

We evaluated phase differences that could contribute to unidirectional information transmission based on the communization-through-coherence theory^4^. UCI of region $n$ was defined by:

$${U}_{j}\left(t\right)=\frac{1}{N}\sum_{k=1}^{N}(0.5\mathrm{cos}\left({\theta }_{j}-{\theta }_{k}-\frac{2\pi {\tau }_{jk}}{{f}_{0}}\right)+0.5)$$

where ${U}_{j}\left(t\right)$ indicates the average communication efficiency from region n at time t with a value ranging from 0 to 1 (the larger the value, the more efficient the communication). In the scale of the entire network, phases appear in sequence regions in accordance with their internal frequencies. This sequencing effect was evaluated using the regression coefficient calculated between internal frequencies and USIs (or slope of USI).

#### Source-sink index (SSI)

The spatial extent of a local radial wave from a region n was measured by:

$$S_{j} (r) = \frac{1}{|K|}\sum\limits_{k \in K} {({\mathbf{x}}_{{\mathbf{k}}} - {\mathbf{x}}_{{\mathbf{j}}} )} \cdot {\mathbf{g}}_{{\mathbf{k},}}$$

where ${\mathbf{x}}_{{\mathbf{k}}}$ denotes the location of region $k$ in the cortical surface map, ${\mathbf{g}}_{{\mathbf{k}}}$ denotes the local phase gradient at ${\mathbf{x}}_{{\mathbf{k}}}$, $K$ denotes the set of region indices satisfying $(r - \Delta r) < |{\mathbf{x}}_{{\mathbf{p}}} - {\mathbf{x}}_{{\mathbf{n}}} | < (r + \Delta r)$, and $r$ denotes the radius from the center of region $j$. The dependency of SSI on the internal frequency was measured using a regression coefficient calculated between the internal frequencies and SSIs (or the slope of SSI).

#### Order parameter

To evaluate the global synchronization of the entire network, we evaluated the order parameter as follows:

$$R\left(t\right)=|\frac{1}{N}\sum_{j=1}^{N}{e}^{i{\theta }_{j}\left(t\right)}|$$

This measure has been frequently used in previous studies^35–39,44^; thus, it was possible to consider the dynamic state of the current model in association with previous studies.

## Supplementary Information

Supplementary Video 1.

Supplementary Information 1.

## Data Availability

All relevant data are within the paper and the codes are available at https://www.fun.ac.jp/~satonao/TravelingWaveKuramoto.zip.
