# Supplementary material for: Cortical traveling waves reflect state-dependent hierarchical sequencing of local regions in the human connectome network
Source: Sci Rep. 2022 Jan 10;12:334. doi: 10.1038/s41598-021-04169-9 (PMC8748796; doi:10.1038/s41598-021-04169-9)
Supplement: Supplementary file 2 — Supplementary Information 1. [file 41598_2021_4169_MOESM2_ESM.docx]

Video: Traveling waves on the cortical surface and gyri surface. The filled circles denote the locations of regions and the colors represent their phases. The color scale and the axes are identical with Fig 2.
